# The Influence of Orthogeriatric Co-Management on Clinical Outcomes After Treatment of Proximal Femoral Fractures—Real World Data of Comparable Cohorts Originating from the Same Geographic Area

**DOI:** 10.3390/jcm14217464

**Published:** 2025-10-22

**Authors:** Manuel Känel, Samuel Känel, Method Kabelitz, Kim Aggeler, Michael Dietrich

**Affiliations:** 1 Department of Health Sciences and Technology, Eidgenössische Technische Hochschule Zürich, 8092 Zurich, Switzerland; manuelk1298@hotmail.com (M.K.); samuelk1298@hotmail.com (S.K.); 2 Clinic for Orthopaedics, Hand- and Trauma Surgery, Stadtspital Zürich, 8037 Zurich, Switzerland; kim.aggeler@stadtspital.ch (K.A.); michael.dietrich@stadtspital.ch (M.D.)

**Keywords:** orthogeriatric co-management, proximal femoral fracture, hip fracture, frailty, outcomes

## Abstract

**Background**: Surgically treated proximal femoral fractures in geriatric patients are a major debilitating condition, with continuously rising numbers, impacting patients and the healthcare system. Models of care based on orthogeriatric co-management (OGCM) have suggested promising clinical outcomes compared with the standard of care (SOC) model in the treatment of frail elderly patients. **Methods**: A retrospective cohort study investigating clinical outcomes in two comparable cohorts of patients aged 75 and older, who underwent surgical treatment for proximal femoral fractures in 2023 was conducted. Included individuals all originated from the same geographic area, therefore presenting a unique cohort. The cohorts were differentiated by the perioperative care protocols implemented: an OGCM protocol (*n* = 147) versus a SOC protocol (*n* = 143). The main outcome measures were readmission, revision, and mortality rates at 30 days and one-year post-surgery, as well as the length of hospital stay. **Results**: Findings revealed a positive impact for patients treated under the OGCM protocol, with a significant reduction in the length of hospital stay (6 vs. 7 days, *p* = 0.001), while no consistent differences were observed in readmission (36.2% vs. 39.7%, *p* = 0.676), surgical revision (8.4% vs. 12.4%, *p* = 0.485), and mortality (24.1% vs. 31.7%, *p* = 0.781) rates one-year after surgery. **Conclusions**: Despite the absence of significant differences in major outcomes such as mortality, readmission, and surgical revision between the two protocols, the implementation of a more resource-intensive multidisciplinary care pathway resulted in a significant reduction in hospital length of stay. Beyond its clinical value, this improvement may contribute to reducing the burden on healthcare staff and support the sustainability of hospital systems facing increasing pressure.

## 1. Introduction

Proximal femoral fractures in the elderly population are a major health concern, with severe impacts on patients’ health, well-being, and independence [1,2]. Worldwide, as well as in Switzerland, the number of proximal femoral fractures is constantly rising, with estimates indicating that by 2050, there will be around 6.3 million fractures annually across the globe [3,4]. This type of fracture represents one of the most serious fall-related injuries, which, driven by demographic shifts, are occurring more frequently in elderly and frail individuals [5,6]. For the treatment of this population, it is important to recognise that fractures constitute only one of several medical challenges, occurring alongside comorbidities, re-hospitalisations, and a one-year mortality rate ranging from 14% to 36% [7,8]. This clinical condition not only has consequences for patients, but also affects their relatives, policymakers, and the healthcare system [9,10,11]. As the number of personnel required for patient care continues to rise, both during hospitalisation and in rehabilitation facilities or nursing homes, the entire care pathway is putting increasing pressure on the healthcare system [12,13].

In order to best manage frail geriatric patients with comorbidities, polypharmacy, and cognitive impairment, who do not only need treatment by an orthopaedic surgeon, several models of orthogeriatric co-management (OGCM) have been developed in the UK since the 1950s [1,14,15].

In recent years, several comparative studies have been carried out to compare standard care pathways and OGCM, in which differences in mortality, re-hospitalisation, length of hospital stay (LOS), and quality of life were analysed [3,16]. Depending on the OGCM model analysed, differences can be observed in the composition of the responsible multidisciplinary teams, as well as in how patient responsibility and management are shared between the geriatrician and orthopaedic surgeon [1,15]. As previously mentioned, models designed for the treatment of patients with complex needs have shown the potential to significantly enhance clinical outcomes [16,17]. These results are made possible by optimising the patients’ perioperative pathways, preventing complications like delirium, through accurate patient assessment and careful management of comorbidities [3,8].

Despite the potential advantages of OGCM, data is still lacking about the superiority of any specific treatment model. Comparisons were frequently performed between noncomparable and heterogeneous cohorts of different sizes, originating from different geographic regions [1,18]. Furthermore, it remains unclear which model is the best one concerning the treatment of geriatric patients with proximal femoral fractures and what are the real benefits of an interdisciplinary model involving the figure of the geriatrician, along with the presence of other specialists [5,19]. Consequently, these studies do not allow identification of the true potential of multidisciplinary care models, given the influence of disturbing factors of different natures.

There are no studies in the literature comparing two hospital centres belonging to a single institution within one unique geographic area, implementing two different well-established protocols for the treatment of proximal femoral fractures. This research aimed to assess the effectiveness of a multidisciplinary OGCM on improving clinical outcomes in frail geriatric patients with proximal femoral fractures. The goal of this study was to investigate whether implementing an OGCM model, instead of a standard of care (SOC) one, could reduce readmission, revision, and mortality rates at 30 days and one-year post-surgery, as well as shorten the LOS.

## 2. Materials and Methods

### 2.1. Study Design

Patients admitted between January and December 2023 to two different sites belonging to the same hospital were identified according to the inclusion criteria for this retrospective cohort study. Patients were consecutively enrolled in the study if they were either above 80 years of age or older than 75 years with at least two major secondary diagnoses. Additional inclusion criteria included the occurrence of low-impact trauma, an operatively treated proximal femoral fracture (femoral neck or trochanteric), and signed informed consent obtained during hospitalisation. Individuals with signs of pathological or atypical fractures and lack of informed consent were excluded.

This study has been conducted in accordance with good clinical practice (GCP) guidelines and the 1964 Declaration of Helsinki and was approved by the local ethics committee (BASEC number 2024-01660).

The two hospital centres considered were centrally administered by a single institution and were geographically separated by a distance of five kilometres. One team of orthopaedic surgeons was responsible for both sites, using similar medical devices and operative techniques. Over the course of a year, the surgical team operatively treated approximately 250 proximal femoral fractures at each site. The two centres differed concerning the perioperative treatment pathway: facility ‘A’ implemented a certified OGCM protocol, whereas facility ‘B’ followed a SOC protocol.

### 2.2. Orthogeriatric Co-Management

The OGCM protocol was based on the shared management of the patient by the geriatrician and the orthopaedic surgeon, deciding by mutual agreement which interventions were most appropriate to be performed during hospitalisation. OGCM was based on a standardised, multidisciplinary patient-oriented care protocol, activated directly after patient admission to the emergency department (ED) if the criteria for inclusion in the OGCM protocol were met, namely, patients aged over 80 years or over 75 years with at least two major secondary diagnoses. Patients treated with an OGCM approach were admitted in the dedicated orthogeriatric ward, where the specific needs and greater complexity of the cases treated were carefully addressed. The geriatrician was a central figure in this protocol, involved in the treatment of the patients from the time of admission, when they were assessed for medication management, comorbidities, and for the presence of neuro-cognitive diseases (for example delirium). At admission, or at the latest postoperatively, three assessments were carried out: the short orientation–memory–concentration (SOMC) test, the Barthel Index, and nutritional risk screening (NRS) [20,21,22]. The hospital course of each individual case was assessed twice a week during multidisciplinary meetings, in which orthopaedic surgeons, geriatricians, nurses, physiotherapists, nutritionists, and social services took part. Each patient received two physiotherapy sessions daily, with the aim of regaining their independence through early mobilisation. According to a standardised schedule, consultations were carried out by nutritionists to assess caloric and protein intake, and when needed, the patients’ nutritional diet was adapted to guarantee daily requirements. From the early stages of hospitalisation, social services were involved in planning each patient’s discharge to the most appropriate facility, adapting plans according to the patient’s hospital course and feedback obtained during multidisciplinary meetings. In addition to the above-mentioned, other specialists such as a psychologist or speech therapist, were included in the hospital care pathway if necessary.

### 2.3. Standard of Care

The SOC protocol implemented in hospital ‘B’ was characterised by the management of the patient led by the orthopaedic surgeon. Patients were admitted to a general ward, with patients of heterogeneous age and diagnosis. The surgeon autonomously decided on the most suitable treatment and timing, evaluated whether to involve other specialists such as nutritionists in the treatment pathway, and determined the most appropriate timing and type of facility for patient discharge. Nearly 30% of patients benefitted from nutritional counselling services, while about 50% received support from social services. The actual planning and organisation of the discharge were then carried out by the hospital’s social services, based on the orthopaedic surgeon’s indications, in those cases where their involvement was required. In this treatment protocol, the geriatrician was involved if deemed necessary by the orthopaedic surgeon, in the form of a clinical consultation. During the hospital stay, patients benefitted from one daily physiotherapy session. The SOMC test, Barthel Index, and NRS assessments were not systematically collected.

### 2.4. Outcome Measures

The primary outcomes of this study were the rates of readmission, revision, and mortality at 30 days and one-year postoperatively, as well as the LOS. Secondary outcomes were the length of stay in rehabilitation institutions (LOS Reha), duration of accompanying services (physiotherapy, nutritional counselling, nursing care), surgical and medical complications, patient surveillance, and the Barthel Index. The LOS Reha was defined as the time period spent in musculoskeletal rehabilitation institutions and specialised geriatric acute rehabilitation. All revision surgeries, as well as non-surgical treatments directly related to the same anatomical area of the fracture, observed up to one-year of follow-up, were defined as surgical complications. Medical complications included all newly diagnosed conditions during the index hospital stay (including delirium; cardiovascular, pulmonary, and renal diagnoses; urinary tract infections (UTIs); and bleeding anaemia). Individual patient surveillance was provided in cases where the patient presented medical instability during the hospital stay, including patients who were disoriented or exhibited delusional behaviour, with a staff member remaining in the room to ensure continuous supervision. The Barthel Index was used to assess patients’ functional independence in activities of daily living, including domains such as feeding, mobility, and bathing.

### 2.5. Data Source

Patients’ data used in the study were retrieved using the local electronic patient information system (KISIM, Cistec AG, Zürich, Switzerland).

### 2.6. Statistical Analysis

The Shapiro–Wilk test was used to test the normality distribution of continuous variables, described with the mean and standard deviation (SD) if they were normally distributed or with the median and interquartile range (IQR) if they were not normally distributed. Binary or categorical variables were described with absolute values and percentages. Differences between the two cohorts for continuous variables were tested with an independent samples T-test or the Wilcoxon signed-rank test, depending on the data distribution. To test differences in the two cohorts for binary or categorical variables, the Chi-square test or Fisher’s exact test were used, depending on the number of observations per category. A *p*-value < 0.05 was considered statistically significant. Statistical analyses were performed using the statistical software R version 4.2.3 (R Core Team, Vienna, Austria).

## 3. Results

**Figure 1:**
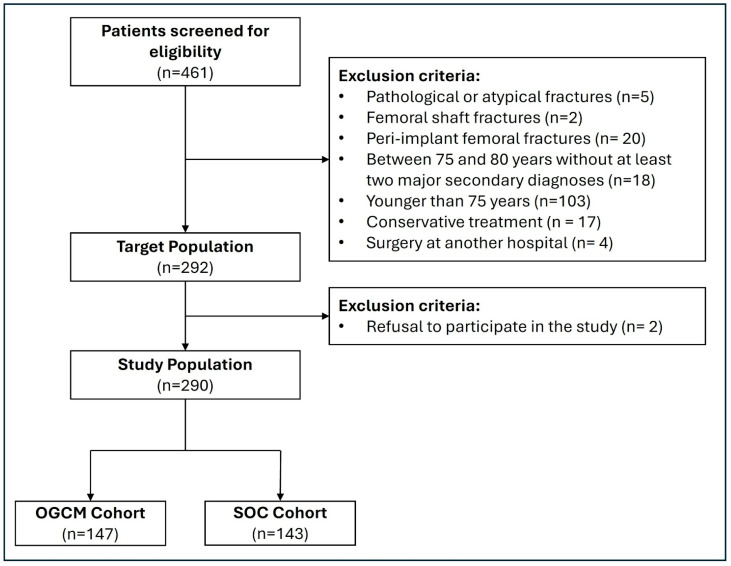
Flowchart of patient inclusion process. SOC = standard of care. OGCM = orthogeriatric co-management.

After screening 461 patients for eligibility, 147 patients were selected for the OGCM protocol and 143 for the SOC protocol for final analysis (Figure 1). In the OGCM cohort, the mean age was 87.5 ± 5.7 years, while in the SOC cohort it was 86.7 ± 6.2 years (*p* = 0.239). Both cohorts had a majority of female patients, accounting for 78.9% (*n* = 116) in the OGCM cohort and 70.6% (*n* = 101) in the SOC one (*p* = 0.136). Additional baseline characteristics of the patients included in the study are presented in Table 1.

**Figure 2:**
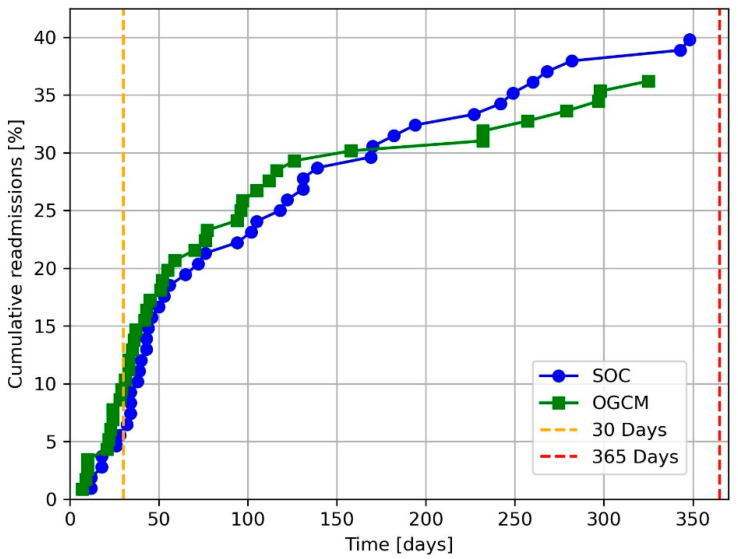
Cumulative readmissions up to one-year post-surgery. SOC = standard of care. OGCM = orthogeriatric co-management.

At 30 days post-surgery, the readmission rate for the OGCM cohort was 8.5% (*n* = 11) and 4.7% (*n* = 6) for the SOC cohort (*p* = 0.324). The rate of readmission after one-year postoperatively was 36.2% (*n* = 42) for the OGCM cohort and 39.8% (*n* = 43) for the SOC one (*p* = 0.676). Figure 2 shows the cumulative readmission rate at one-year for the two cohorts.

**Figure 3:**
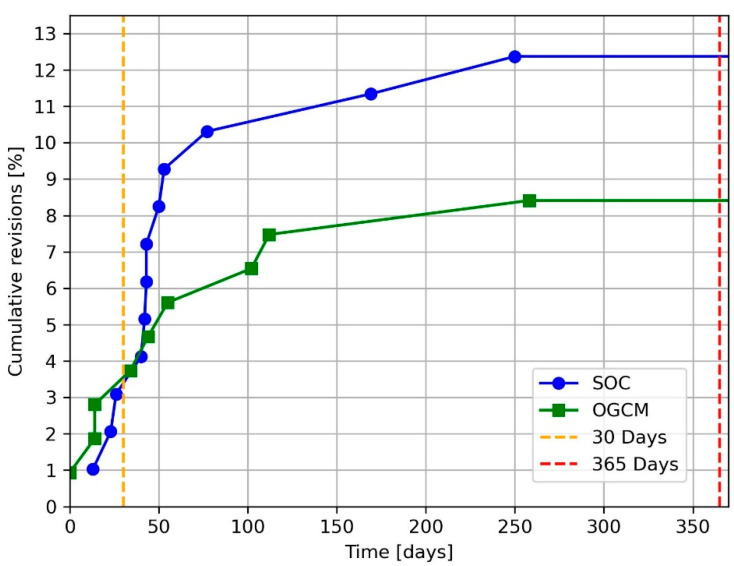
Cumulative revisions up to one-year post-surgery. SOC = standard of care. OGCM = orthogeriatric co-management.

The revision rate at 30 days post-surgery was equivalent for both treatment protocols, with three patients (2.3%) revised per cohort (*p* = 1). One-year post-surgery, 8.4% (*n* = 9) of the patients in the OGCM cohort underwent a revision intervention, compared with 12.4% (*n* = 12) in the SOC cohort (*p* = 0.485). Figure 3 shows the one-year cumulative revision curve for both considered cohorts.

**Figure 4:**
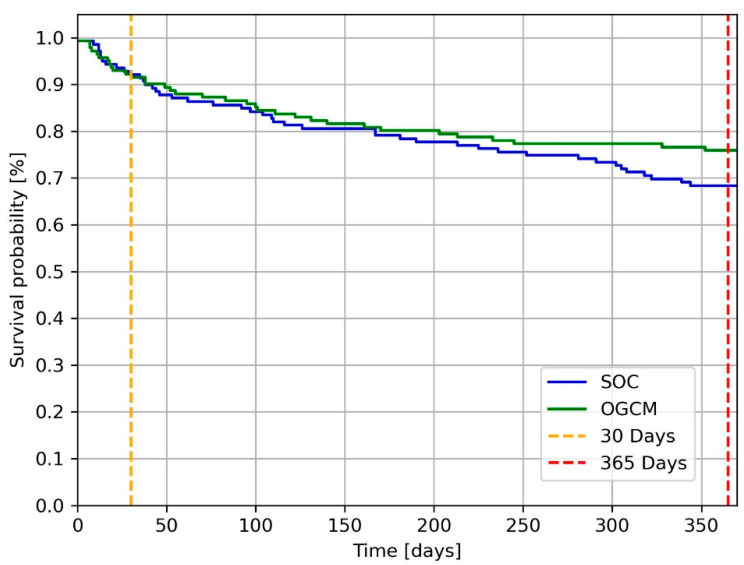
Survival curve illustrated with Kaplan–Meier. At 30 days post-intervention, 12 OGCM patients and 11 SOC patients died. At one-year post-surgery 34 OGCM patients and 44 SOC patients died. SOC = standard of care. OGCM = orthogeriatric co-management.

Considering the mortality at 30 days postoperatively, the OGCM cohort presented a mortality rate of 8.5% (*n* = 12) compared with 7.9% (*n* = 11) in the SOC cohort (*p* = 1). Within the first-year post-surgery, the OGCM protocol reported a mortality rate of 24.1% (*n* = 34) and the SOC protocol that of 31.7% (*n* = 44; *p* = 0.203). In-hospital mortality recorded for the two cohorts was 4.1% (*n* = 6) for OGCM and 2.8% (*n* = 4) for SOC, respectively (*p* = 0.781). Presented in Figure 4 is the one-year survival curve for the two cohorts.

The cohort undergoing the OGCM protocol had a significantly shorter LOS, with a median hospital stay of 6 days (IQR 5–8) compared with 7 days (IQR 6–9) in the SOC cohort (*p* = 0.001).

Analysing the secondary outcomes, LOS Reha was identical for both cohorts, with a median duration of 14 days. Regarding the minutes of physiotherapy received by patients during hospitalisation, the OGCM cohort benefitted from significantly more physiotherapy with a median of 392 min (IQR 283–794) compared with 315 min (IQR 205–630) in the SOC cohort (*p* = 0.007). Similarly, the OGCM cohort received significantly more minutes of nutrition counselling compared with the SOC one, with respective medians of 306 min (IQR 235–449) and 0 min (IQR 0–235) per cohort (*p* < 0.05). Concerning the minutes of nursing care, the OGCM cohort received a median of 3′444 min (IQR 2′461–5′592) compared with 3′724 min (IQR 2′356–5′862) for the SOC cohort (*p* = 0.676). Analysing complications, 54.4% of patients in the OGCM cohort and 65% in the SOC cohort experienced at least one medical complication (*p* = 0.085; Table 2). Considering surgical complications, there were 6.1% of patients in the OGCM cohort and 10.5% in the SOC cohort who were affected (*p* = 0.256). The OGCM cohort required significantly less patient surveillance, with 3% (*n* = 5) compared with 29% (*n* = 41) in the SOC cohort (*p* < 0.05). Between the two cohorts, there was a statistically significant difference for the Barthel Index at time of hospitalisation, with a median score of 25 (IQR 15–40) for the OGCM cohort and 45 (IQR 35–55) for the SOC cohort (*p* < 0.05).

## 4. Discussion

This study evaluated the effect of an OGCM compared with an SOC model of care on the complex treatment of proximal femoral fractures in an elderly and frail population. Multidisciplinary models of care have gained increasing acceptance in the recent past for the treatment of geriatric trauma patients, as a result of their demonstrated ability to improve clinical outcomes and preserve quality of life [3,23]. In the present study, the OGCM pathway showed comparable results to the SOC model in terms of readmission, revision, and mortality rates, but was associated with a significant reduction in LOS.

Readmission rates at 30 days and one-year were not significantly lower in the OGCM cohort compared with the SOC cohort. In the OGCM group, 8.5% of patients were readmitted within 30 days after surgery, compared with 4.7% in the SOC group; both rates were lower than those reported from other studies, where readmission ranged from 10.3% to 11.9% [15,24,25]. Considering the one-year readmission rate, the OGCM reported a rate of 36.2% compared with 39.8% for the SOC. A recent study conducted by Rapp et al. showed an even greater difference, with a readmission rate of 25.7% for OGCM and 39.7% for SOC [3]. This difference could be explained by the exclusion of patients at high surgical risk in the study previously cited, excluding subjects with an ASA score of four or five. As reported by Kates et al. and Rapp et al., medical complications post discharge represent the main reason for readmission [3,25]. The present study, in turn, confirms this result, with medical complications accounting for 83.3% of readmissions in the OGCM cohort and 74.4% in the SOC cohort (Table 2).

With respect to the revision rate, our study found no significant differences either at 30 days or at one-year postoperatively comparing the two cohorts. Unfortunately, it was not possible to compare our results with the literature, due to the lack of studies analysing the impact of OGCM on revision rates.

Analysing the mortality rate at 30 days and one-year postoperatively, no significant difference was found in our study. The OGCM cohort had a 30-day mortality rate equivalent to 8.5% compared with 7.9% in the SOC cohort. In a recent study by Rapp et al., the OGCM had a lower 30-day mortality rate than the SOC, with 10.3% compared with 13.4%, however, showing higher rates than those obtained in the present study [7]. At one-year postoperatively, the OGCM showed a mortality rate of 24.1% compared with 31.7% for the SOC. Our results are consistent with those obtained by Neuerburg et al., reporting a rate of 22.8% for OGCM and 28.1% for SOC [3]. Some studies have obtained greater differences between the two protocols, with lower overall mortality rates, that might be influenced by the inclusion of younger patients, associated with fewer comorbidities and post-surgical complications [1,17,26]. Regarding the in-hospital mortality rate, the OGCM protocol reported a rate of 4.1% and the SOC that of 2.8%, a result comparable with the respective 1.9% and 4.4% obtained by Henderson et al. [26]. It is important to note that country-dependent variations in hospital stay may directly influence the in-hospital mortality rate [1,27]. As suggested by several studies in the literature, lower readmission, and mortality rates seem to be allowed by a comprehensive assessment of patients at admission, enabling better identification and management of existing comorbidities, avoiding preventable complications [13,28,29].

The LOS was significantly shorter for patients treated with OGCM than for those treated with SOC (*p* = 0.001). This result is not homogeneously reflected in the literature, as depending on the healthcare system and the country considered, protocols foresee different LOS [7,17,30]. In contrast to several other studies, we found that the time until surgery was not a key factor influencing the LOS, as for both cohorts, the median time until surgery was comparable and below 24 h (Table 3) [26,30,31]. A shorter hospitalisation, in agreement with the literature, seems to be strongly influenced by fewer complications and by an early planning of the discharge [17,24]. The latter two factors are facilitated by the presence of the geriatrician and the social services.

Regarding the LOS Reha, OGCM revealed no difference compared with SOC. Analogous results were obtained by Boddaert et al., where not only was there no difference concerning the duration of rehabilitation, but similarly to our findings, the hospital stay was also significantly shorter for patients treated by a multidisciplinary team [27]. The LOS Reha is difficult to compare with other studies, since it is rarely analysed due to limited access to such data or because geriatric acute rehabilitation is often integrated during the hospital stay, as is the case in Germany [5,7,9]. As argued by Ho et al. and suggested by our study, a reduction in the LOS did not have a negative influence on the duration of the subsequent rehabilitation phase [32].

Two central factors in OGCM are physiotherapy and nutritionist counselling, which were provided significantly more frequently in the OGCM-treated patients. Although the hospital stay was shorter, more minutes of physiotherapy were provided to the OGCM cohort, given a greater number of physiotherapy sessions, which may have speeded up functional recovery and prepared patients for discharge more quickly [3,33,34]. Similarly, nutritional counselling might have contributed to preventing complications and optimising the nutritional status of patients prior to discharge, allowing for a shorter hospital stay [4,30].

During the hospital stay, patients in the two cohorts benefitted from a comparable number of NCP minutes. However, considering the NCP provided on a daily basis, the OGCM cohort received significantly more minutes than the SOC cohort, with a median of 424 and 398 daily minutes per cohort, respectively (*p* = 0.037). Unfortunately, it was not possible to quantitatively compare the findings with the literature. Nevertheless, a study conducted by Titler et al. reported fewer complications and a shorter LOS by means of a multidisciplinary protocol, characterised by a greater supervision of the nursing staff [10].

As previously highlighted, complications play a crucial role for the clinical outcomes, including readmission, revision, and mortality. The majority of diagnosed complications in both cohorts were of medical nature, with 65% of patients in the SOC cohort and 54.4% in the OGCM cohort experiencing at least one medical complication. This result was also highlighted in several studies, which reported that medical complications were more frequent than surgical ones, with both being more prevalent in the SOC cohort [19,24,35]. Delirium emerged as one of the most prominent medical complications in our study, diagnosed in 37.4% of OGCM patients and 46.9% of SOC patients. Although it was not possible to compare the occurrence of delirium in the respective cohorts with the literature, a recent study by Folbert et al. reported that 24.4% of the patients were diagnosed with delirium [19]. An interesting finding related to complications concerns the performance of patient surveillance, a service provided to medically or psychologically (e.g., delirium) unstable individuals. In the present study, 5 patients in the OGCM cohort required this service compared with 41 in the SOC cohort. Unfortunately, no studies analysing patient surveillance were identified in the literature, making a comparison impossible. Our findings align with those of previous studies, suggesting that a comprehensive patient assessment at admission and the inclusion of the geriatrician as a central figure in the multidisciplinary team help to limit the occurrence of medical complications such as delirium, thereby contributing to decreasing the LOS [3,17,28].

Concerning activities of daily living, the cohort treated with OGCM had a significantly lower Barthel Index score (*p* < 0.05). As suggested by Folbert et al., a low Barthel Index represents a risk factor for the occurrence of adverse events like postoperative complications [19]. However, direct comparisons between cohorts is limited, as the Barthel Index was routinely assessed only in the OGCM cohort and was available for fewer than half of the patients (*n* = 59) in the SOC cohort. Additionally, we were unable to compare the scores of our cohorts due to the lack of studies comparing the Barthel Index at admission.

To the best of our knowledge, the present study is the first in Switzerland to have investigated the impact of an OGCM compared with a SOC model, analysing two contemporaneous patient cohorts from the same metropolitan area treated according to two well-established care protocols. Unlike many studies in the literature, both analysed cohorts included patients originating from one single metropolitan area, exposed to the same environmental and socio-economic factors, thus eliminating many confounding factors [5,7]. Another strength of this study lies in the comparison of two hospital sites with two different, well-established treatment protocols active during the same historical period. In contrast, many studies compared OGCM cohorts to historical control groups [19,26], with sometimes multidisciplinary treatment protocols investigated during the implementation phase [1,23].

On the other hand, this retrospective cohort study has several limitations that should be considered when interpreting the findings. The OGCM cohort consisted of less healthy patients, as indicated by a lower Barthel Index score and a higher proportion of subjects from nursing homes. Therefore, a dampening of the possible positive effects promoted by an OGCM protocol cannot be excluded. A second limitation concerns the relatively small cohort sizes, for which we were unable to find significant differences in readmission, revision, and mortality rates at one-year post-surgery, although several studies have reported significant benefits in favour of the OGCM protocol. A further weakness is the retrospective nature of our study. However, direct access to all collected patient data and the allocation of patients to one hospital site or the other, according to hospital capacity, mitigated this limitation. Care must be taken when comparing the results obtained in our study with those in the literature, as the findings may be influenced by the organisation of the healthcare system in place in different countries.

## 5. Conclusions

The ongoing increase in proximal femoral fractures among the frail and geriatric population has raised serious concerns for the healthcare system. The present study of a unique cohort attempted to investigate the influence of the OGCM care pathway on geriatric patients with proximal femoral fractures. In this study, no significant differences were observed between OGCM and SOC in terms of readmission, revision, and mortality rates, indicating that the OGCM protocol was not inferior with respect to these key outcomes. Nevertheless, the introduction of a more resource-intensive multidisciplinary OGCM protocol resulted in a significant reduction in hospital length of stay. This outcome is of particular clinical importance, as it allows patients to be discharged earlier and to start rehabilitation sooner, while simultaneously reducing the workload of healthcare personnel and the burden on hospitals in a context of growing systemic pressure. These results support the rationale for further exploring and refining structured multidisciplinary care models in the management of frail elderly patients with proximal femoral fractures.

## Figures and Tables

**Table 1:** Baseline and demographic characteristics.

Characteristic	OGCM (*n* = 147)	SOC (*n* = 143)	*p*-Value
Age [years], mean (± SD)	87.5 (5.7)	86.7 (6.2)	0.239
Women, *n* (%)	116 (78.9)	101 (70.6)	0.136
BMI [kg/m^2^], mean (± SD)	23.1 (4.2)	23.8 (5.7)	0.213
Fracture type, *n* (%)			
Trochanteric	73 (49.7)	80 (55.9)	0.340
Femoral neck	74 (50.3)	63 (44.1)	0.340
Left side fracture, *n* (%)	71 (48.3)	56 (39.2)	0.147
Residence before admission, *n* (%)			
Home	85 (57.8)	86 (60.1)	0.778
Retirement home	14 (9.5)	27 (18.9)	0.034
Nursing home	48 (32.7)	29 (20.3)	0.024
Other hospital	0 (0)	1 (0.7)	0.989
Mobility before admission, *n* (%)			
Independent	55 (38.2)	51 (35.9)	0.782
Stick	19 (13.2)	21 (14.8)	0.827
Rollator	52 (36.1)	56 (39.4)	0.647
Walker	0 (0)	1 (0.7)	0.994
Helping person	4 (2.8)	2 (1.4)	0.693
Wheelchair	14 (9.7)	11 (7.8)	0.702
Barthel Index [0–100], mean (± SD)	30 (17.4)	45.9 (19.8)	<0.05
SOMC Test [0–28], mean (± SD)	13.7 (10.1)	10.2 (8)	0.033
NRS [0–7], mean (± SD)	3.4 (1)	3 (1.3)	0.072
ASA classification [1–6], *n* (%)			
2	23 (15.7)	33 (23.1)	0.146
3	111 (75.5)	98 (68.5)	0.233
4	13 (8.8)	12 (8.4)	1
Antithrombotic agents, *n* (%)	78 (53.1)	83 (58)	0.462
Antiosteoporotic medication, *n* (%)	89 (60.5)	63 (44.1)	0.007

SD = standard deviation; *n* = number of patients; SOC = standard of care; OGCM = orthogeriatric co-management; BMI = Body Mass Index; SOMC = short orientation–memory–concentration; NRS = nutritional risk screening; ASA = American Society of Anaesthesiologists Classification; antithrombotic agents included platelet aggregation inhibitors and antithrombotic medications; Barthel Index, SOMC, and NRS assessments were available for only 59, 35, and 68 patients, respectively, in the SOC cohort.; *p*-value < 0.05 was considered statistically significant.

**Table 2:** Medical complications.

Complication	OGCM (*n* = 147)	SOC (*n* = 143)
Delirium, *n* (%)	55 (37.4)	67 (46.9)
Cardiovascular, *n* (%)	10 (6.8)	9 (6.3)
Pulmonary, *n* (%)	13 (8.8)	11 (7.7)
Urinary tract infection, *n* (%)	19 (12.9)	13 (9.1)
Bleeding anaemia, *n* (%)	3 (2)	41 (28.7)
Renal insufficiency, *n* (%)	6 (4.1)	4 (2.8)
Systemic infection, *n* (%)	1 (0.7)	3 (2.1)
Liver failure, *n* (%)	1 (0.7)	0 (0)
Opiate overdose, *n* (%)	1 (0.7)	0 (0)
Colitis, *n* (%)	1 (0.7)	0 (0)
Stroke, *n* (%)	0 (0)	1 (0.7)

*n* = number of patients; SOC = standard of care; OGCM = orthogeriatric co-management.

**Table 3:** Outcome measures.

Outcome	OGCM (*n* = 147)	SOC (*n* = 143)	*p*-Value
Readmission reason at 1 year, *n* (%)			
Medical	35 (83.3)	32 (74.4)	0.459
Surgical	7 (16.7)	11 (25.6)	0.459
Time to surgery [h], median (IQR)	16.3 (7–27)	15.3 (10–23)	0.939
Residence after discharge, *n* (%)			
Home	3 (2)	4 (2.8)	0.971
Retirement home	9 (6.1)	21 (14.7)	0.028
Nursing home	50 (34)	30 (21)	0.019
Geriatric rehabilitation	46 (31.3)	66 (46.2)	0.013
Musculoskeletal rehabilitation	33 (22.4)	18 (12.6)	0.040
Haemoglobin level [g/L], median (IQR)			
Before surgery	124 (112–131)	120 (110–130)	0.151
After surgery	98 (87–110)	93 (83–106)	0.023
Blood infusion, *n* (%)	31 (21.1)	51 (35.7)	0.009

IQR = interquartile range; *n* = number of patients; SOC = standard of care; OGCM = orthogeriatric co-management; *p*-value < 0.05 was considered statistically significant.

## Data Availability

Anonymised participant data will be made available upon reasonable requests directed to the corresponding author.

## Abbreviations

The following abbreviations are used in this manuscript:

OGCM	Orthogeriatric co-management
SOC	Standard of care
LOS	Length of stay
LOS Reha	Length of stay in rehabilitation institutions
GCP	Good clinical practice
ICU	Intensive care unit
ED	Emergency department
UTI	Urinary tract infections
SOMC	Short orientation–memory–concentration
NRS	Nutritional risk screening
IQR	Interquartile range
SD	Standard Deviation
